# Estimating Tumor Proportion in Smear Slides for Reliable Molecular Analysis

**DOI:** 10.5146/tjpath.2026.13923

**Published:** 2026-01-31

**Authors:** Cisel Aydın Mericoz, Ibrahim Kulac, Pinar Fırat

**Affiliations:** Department of Pathology, School of Medicine, Koç University, Istanbul, Türkiye; Department of Pathology, School of Medicine, Koç University, Istanbul, Türkiye; KUIS Artificial Intelligence Center, Koç University, Istanbul, Türkiye; Research Center for Translational Medicine, Koç University, Istanbul, Türkiye

**Keywords:** Tumor proportion, Cytology smears, Molecular analysis

## Abstract

**Objective:** The use of molecular pathology is critical in diagnostics and theranostics. Today, cytological smears are utilized for molecular testing more often than ever. Accurate tumor cell percentage estimation is essential for reliable molecular testing, but its consistency remains uncertain. This study evaluates the reliability of tumor cell percentage estimations among an expert cytopathologist, a molecular cytopathologist, and a molecular pathologist.

**Material and Methods:** Digital images from May-Grünwald-Giemsa (MGG)-stained smear slides of ten EBUS-guided mediastinal lymph node samples were selected. Five regions per slide were evaluated (50 areas from 10 patients). Three pathologists independently estimated tumor cell percentages using predefined categories (0–10%, 11–20%, 21–50%, etc.). Cells were also counted manually as the gold standard.

**Results:** The molecular cytopathologist (Observer 1 -) showed the highest consistency (Kappa = 0.69), followed by the expert cytopathologist (Observer 3 -, Kappa = 0.64), both demonstrating substantial agreement with the gold standard. The molecular pathologist (Observer 2 -) displayed moderate consistency (Kappa = 0.52). Agreement was most significant in the 71–100% category, aligning in over 95% of cases. The lowest value occurred in the 11–20% category. In this category, tumor proportions were frequently overestimated compared to the gold standard.

**Conclusion:** Variability in tumor percentage estimations shows the need for standardized protocols and training. Substantial agreement was reached in specific categories. However, discrepancies in borderline cases highlight the importance of accurate assessments. More research is needed to improve estimation methods.

## 
INTRODUCTION

Precision medicine has changed how we approach cancer patients. Molecular pathology is central in identifying genetic alterations that can be targeted therapeutically. Next-Generation Sequencing (NGS) has made it much easier to analyze tumors’ genetic and transcriptomic features. Still, its utility largely depends on factors like tumor cell ratio and specimen quality. Detecting actionable mutations in non-small cell lung cancer (NSCLC) is a classical example (1). Introducing tyrosine kinase inhibitors has changed how we evaluate and treat lung cancer patients. However, the effectiveness of these targeted therapies depends on correctly identifying these alterations. This can be challenging especially in specimens with low tumor content (2,3).

Most metastatic NSCLC cases are diagnosed based on endoscopic ultrasound (EBUS) -guided mediastinal lymph node sampling (4). Because of the technique’s inherent difficulties, cytological material usually harbors lower tumor content and ratio than surgical resection specimens. Despite these disadvantages, cytological samples are used in NGS-based studies, and guidelines recommend cytological material with sufficient tumor cell content (5–7).

Although the importance of tumor content in molecular pathology tests is well recognized, there is still limited data on the consistency and accuracy of these estimations in cytologic samples. Our study aims to evaluate the consistency of tumor cell percentage estimations among cytopathologists and surgical pathologists and assess the impact of these estimations on molecular testing. We aim to contribute to methodologies that enhance the reliability of molecular diagnostics and improve patient care.

## MATERIAL AND METHODS

### Digital Imaging

The study included ten smear slides from NSCLC mediastinal lymph node EBUS samples. Slides were stained with May-Grünwald-Giemsa (MGG) and digitized at 20x magnification using a Philips IntelliSite UltraFast scanner (Koninklijke Philips, Amsterdam, Netherlands). Five distinct areas of each slide (n=50 in total) were captured for analysis. Each captured area measured 700 x 1,000 pixels on average, with slight variations depending on the field of interest.

### Pathologist Assessment

Three pathologists independently estimated the tumor cell percentage in each captured area using predefined interval categories: 0–10%, 11–20%, 21–50%, 51–70%, and 71–100%. The first pathologist (observer 1), experienced in molecular pathology and cytopathology, had ten years of experience. The second pathologist (observer 2) specialized in neuropathology and molecular pathology, with over ten years of experience. The third (observer 3) was a senior consultant in cytopathology with over 20 years of experience. All three observers made their initial estimations by visual inspection (“eyeballing”) of digital images, typically spending less than 30 seconds per field.

After these rapid assessments, experienced cytopathologists (observer 3) printed the images and performed detailed manual counts to establish the gold standard. For each field, all intact and evaluable nucleated cells were included. Degenerated, crushed, or morphologically unclassifiable nuclei were excluded. Non-tumor elements such as lymphocytes and background cells were counted in the denominator to maintain consistency.

Tumor proportion was calculated by dividing the number of tumor cells by the total number of intact nucleated cells. On average, 84 cells (range: 18–439) were counted per field, with a median of 71 cells. Tumor percentages ranged from 0.9% to 98.4%, with a mean of 47.1%. These detailed counts served as the reference standard for validating observer estimations.

### Statistical Analysis

Cohen’s Kappa (κ) coefficient was used to measure the level of agreement between each observer and the gold standard across categorical tumor proportion estimates. Kappa values were interpreted as follows: <0.20 poor, 0.21–0.40 fair, 0.41–0.60 moderate, 0.61–0.80 substantial, and >0.80 almost perfect agreement. In addition to the Kappa coefficients, corresponding 95% confidence intervals and p-values were calculated to assess the statistical significance and precision of the agreement. All statistical analyses were performed using IBM SPSS Statistics for Windows, version 28.0 (IBM Corp., Armonk, NY, USA), and a p-value of <0.05 was considered statistically significant. The Kappa results, including confidence intervals and significance values, are presented in detail in the Results section.

## RESULTS

Cohen’s Kappa analysis revealed varying levels of agreement between each observer and the gold standard. Observer 1 demonstrated the highest agreement, with a Kappa value of 0.69 (95% CI: 0.532–0.842, p < 0.001). Observer 3 also showed substantial agreement, with a Kappa of 0.64 (95% CI: 0.479–0.797, p < 0.001). Observer 2, a molecular pathologist without cytopathology training, had a moderate level of agreement, with a Kappa of 0.52 (95% CI: 0.328–0.704, p < 0.001). These results emphasize the impact of cytopathology expertise on tumor proportion estimation in smear slides and support the need for standardized training and consensus-based evaluation in molecular pathology workflows. Among the categories, the highest agreement was observed in the 71–100% category, where all observers (observer 1, observer 2, observer 3) and the gold standard aligned in over 95% of the cases. In contrast, the lowest agreement was noted in the 11–20% category, where differences in tumor proportion estimations were higher than the gold standard, particularly in borderline cases.

### Category Analysis

Below are the detailed agreement results between observers after splitting tumor cell ratios (defined by the gold standard) into categories.

### 0–10% Category

**Figure 1:**
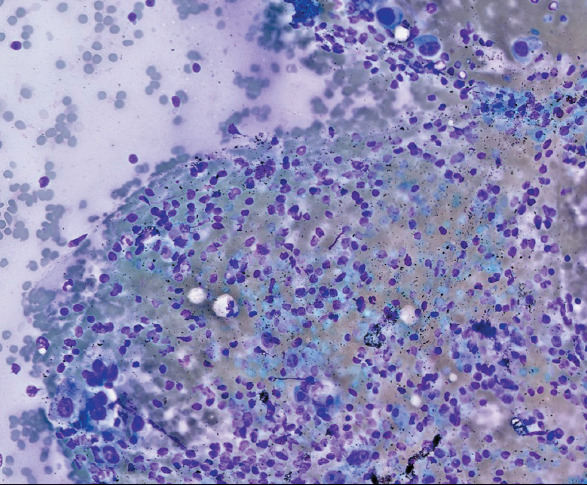
Tumor proportion by gold standard: 0–10%. (Observer evaluations: Observer 1: 0–10%, Observer 2: 0–10%, Observer 3: 0–10%).

Agreement was high in this category. Observer 1 and Observer 2 matched the gold standard in 5 out of 6 fields (83%), while Observer 3 achieved perfect concordance in all 6 fields (100%). This consistency highlights the ease of classification for low tumor proportions (Figure 1).

### 11–20% Category

**Figure 2:**
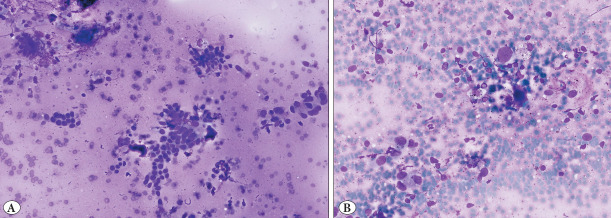
Tumor proportion by gold standard: 11–20%. (2a Observer evaluations: Observer 1: 21-50%, Observer 2: 11–20%, Observer 3: 11-20%) (2b Observer evaluations: Observer 1: 11–20%, Observer 2: 21–50%, Observer 3: 11–20%).

Agreement in this category was moderate. Observer 1 and Observer 2 each aligned with the gold standard in 4 out of 6 fields (67%), while Observer 3 matched in 3 out of 6 (50%) (Figure 2).

### 21–50% Category

**Figure 3:**
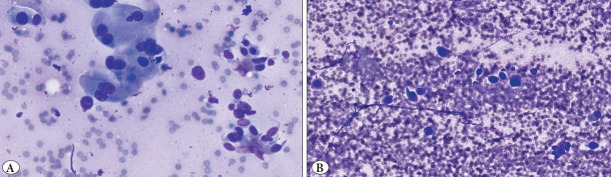
Tumor proportion by gold standard: 21–50%. (3a Observer evaluations: Observer 1: 21–50%, Observer 2: 21–50%, Observer 3: 21–50%) (3b Observer evaluations: Observer 1: 51–70%, Observer 2: 51–70%, Observer 3: 21–50%).

Observer 3 showed the highest agreement in this category, matching the gold standard in 13 of 16 fields (81%). Observer 1 and Observer 2 each correctly classified 11 of 16 fields (69%) (Figure 3).

### 51–70% Category

**Figure 4:**
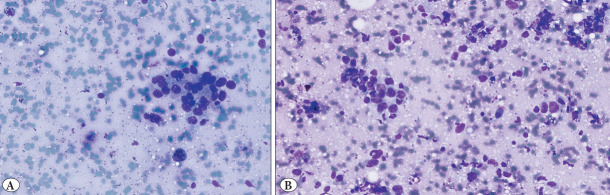
Tumor proportion by gold standard: 51–70%. (4a Observer evaluations: Observer 1: 71–100%, Observer 2: 51–70%, Observer 3: 71–100%) (4b Observer evaluations: Observer 1: 51–70%, Observer 2: 21–50%, Observer 3: 71–100%).

Agreement was more variable. Observer 1 correctly identified 11 of 15 fields (73%), while Observer 2 and Observer 3 matched the gold standard in 8 (53%) and 7 (47%) fields, respectively (Figure 4).

### 71–100% Category

**Figure 5:**
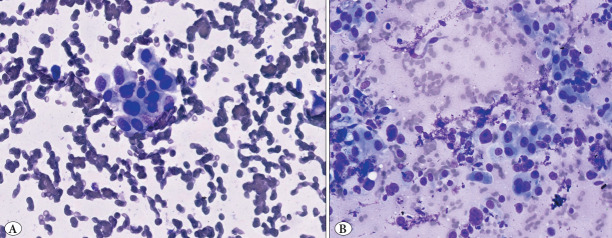
Tumor proportion by gold standard: 71–100%. (5a Observer evaluations: Observer 1: 71–100%, Observer 2: 71–100%, Observer 3: 71–100%) (4b Observer evaluations: Observer 1: 71–100%, Observer 2: 51–70%, Observer 3: 71–100%).

This category demonstrated the highest consistency for Observers 1 and 3, both of whom correctly classified all 7 fields (100%). Observer 2 aligned with the gold standard in 4 of 7 fields (57%) (Figure 5).

### Lowest Agreement

**Figure 6:**
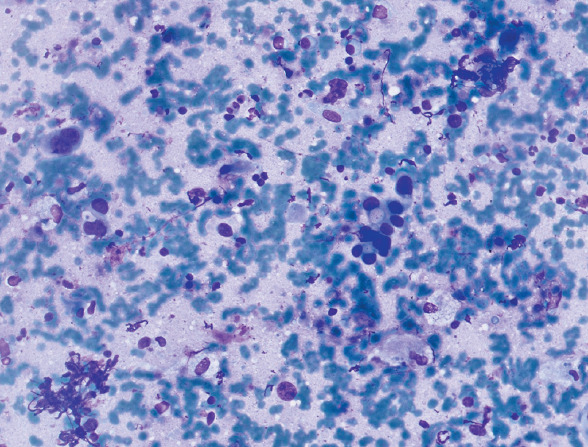
Lowest agreement, tumor proportion by gold standard: 11–20%. (Observer 1 evaluations: 0–10%, Observer 2 and Observer 3:21–50%).

The lowest agreement was observed in fields with mixed cell types and borderline tumor proportions. An example is Figure 6, where the gold standard categorized the tumor proportion as 11–20%, but Observer 1 assessed it as 0–10%, and Observer 2 and Observer 3 as 21–50%. This variability emphasizes the challenges in ambiguous fields (Figure 6).

## DISCUSSION

Estimating tumor cell percentage in samples for molecular testing is a crucial step that directly impacts the reliability of test results. The variation in these assessments across different pathologists highlights the need for standardized methods. This becomes particularly important when the estimated tumor percentage approaches the threshold required for reliable mutation detection, and an overestimation due to insufficient tumor DNA and too much normal DNA contamination from non-tumor cells can lead to false-negative results (2,8,9).

Studies have shown that estimation of tumor ratio may vary between observers (3,10). This may potentially cause serious problems. Overestimating tumor ratio above 20% may cause a false negativity while underestimation may result in unnecessary re-biopsy (11,12). These findings suggest that even experienced pathologists could benefit from trainings focused on tumor estimation.

The highest observer agreement was in the 71–100% category, probably because of the ease of recognizing high tumor proportions. On the other hand, the 11–20% category demonstrated the lowest agreement, emphasizing the difficulty in borderline cases. Overestimation in the 11-20% category is another problem as it is the borderline category and has the potential of false negative results.

The observers in our study came from different backgrounds; observer 1, a cytopathologist with experience in molecular pathology; observer 2, a surgical pathologist with experience in molecular pathology; observer 3, a consultant cytopathologist. Observer 1, no surprise, had the highest concordance whereas observer 2 had the lowest. This shows that cytopathology expertise is critical in tumor proportion assessment in cytology samples. Molecular pathology laboratories should include cytopathologists in their workflow in order to achieve better results. Direct communication between molecular pathologists and cytopathologists would also improve tumor ratio estimation, and thus test accuracy.

Pathologists should consider taking a more conservative approach in their estimations, using tissue macrodissection or microdissection techniques when needed (13–15), and staying informed about the sensitivity of the assays performed in their labs. Standardized trainings that focus on cytological sample evaluation, including workshops, could significantly improve interobserver agreement.

The tumor percentage in the existing material is crucial in selecting the appropriate test for molecular analysis. If the tumor percentage is too low for NGS, other options like immunohistochemistry, FISH, or single-gene analysis can be considered. The integration of NGS and digital PCR, holds the potential to overcome some of these issues by detecting mutations in very small fractions of tumor cells (16). In cases where the number of tumor cells is not adequate, ctDNA analysis using peripheral blood might be an alternative. AI-driven image analysis tools could also help improve consistency.

While this study provides valuable insights, there are some limitations. The gold standard was established through manual cell counting, but distinguishing between certain cells (e.g., small lymphocytes vs. tumor cells) was not always straightforward. This could introduce minor inaccuracies in the gold standard, potentially affecting its reliability as a reference. Notably, the expert cytopathologist who determined the gold standard had a Kappa value of 0.64 in her estimations, showing substantial but not perfect agreement with her own manual counts. This underscores the challenge of achieving absolute precision, even for highly experienced pathologists.

Additionally, the study was limited to 50 fields from 10 cytological slides. While this was enough to capture inter-observer variability and agreement trends, a larger dataset with more diverse specimen types would improve the generalizability of these findings. Lastly, although the observers worked independently, their familiarity with everyday challenges in cytological evaluation might have introduced some subtle biases in their estimations

In fact, for whole-slide assessment or for defining the precise area to be scraped for molecular studies, performing manual counting on a printed version of the selected area would theoretically yield the most accurate results. However, since this is not feasible in routine practice, an alternative approach could be recommended: selecting and printing the area that most closely matches the visually estimated tumor-rich region, and performing manual counting on this printed area similar to the approach used in Ki-67 evaluation (17). This practical strategy may help bridge the gap between ideal and real-world workflows.

Future studies should include larger case numbers and a more diverse set of observers to confirm the generalizability of our findings and support the development of standardized evaluation protocols.

Approaches such as multiplexed imaging and AI-based counting systems have the potential to improve the accuracy of tumor proportion assessments significantly. Incorporating these technologies into routine workflows could also help standardize evaluations across different pathology laboratories. Recent advances in artificial intelligence and intense learning-based tools have demonstrated promising results in tumor cell detection and quantification in non-gynecologic cytology specimens, offering reproducibility and scalability in diagnostic workflows (18–21). As these systems become more accessible and better integrated, they may help overcome current challenges related to interobserver variability and subjective estimation.

## Ethical Approval

This study was performed in accordance with the Declaration of Helsinki.

## Conflict of Interest

The authors declare that they have no conflict of interest to disclose.  

## Funding

No funding received.
